# Motives for alcohol use, risky drinking patterns and harm reduction practices among people who experience homelessness and alcohol dependence in Montreal

**DOI:** 10.1186/s12954-023-00757-2

**Published:** 2023-02-24

**Authors:** Rossio Motta-Ochoa, Natalia Incio-Serra, Alexandre Brulotte, Jorge Flores-Aranda

**Affiliations:** 1 École de travail social, Université du Québec à Montréal, 455 René-Lévesque Blvd. Est Local W-4020, Montreal, QC H2L 4Y2 Canada; 2 École de travail social, Université de Montréal, Pavillon Lionel-Groulx, 3150 Jean-Brillant Street (C-7075), Montreal, QC H3T 1J7 Canada; 3 Canada Research Chair in Sexually and Gender Diverse Individuals (SGD) and Their Psychoactive Substance Use Trajectories (TRADIS Chair), Montreal, Canada; 4 Faculty of Education, McGill University, 3700 McTavish Street, Montreal, QC H3A 1Y2 Canada

**Keywords:** Motives for alcohol use, Patterns of alcohol use, Harm reduction, People who experience homelessness, Alcohol dependence, Risk management

## Abstract

**Background:**

People experiencing homelessness are disproportionately affected by harms related to alcohol use. Indeed, their alcohol dependence is associated with numerous physical and mental health problems along with strikingly high rates of alcohol-related mortality. Recent research has extensively examined alcohol use patterns among people experiencing homelessness in an effort to develop interventions and treatments for this problem. However, only a few studies have incorporated the perspectives of the individuals under study about their drinking or examined the ways in which they manage the associated harms. To bridge this gap, we conducted a qualitative study exploring the relation between the drinking motives, risky drinking patterns and harm reduction practices of a group of people (*n* = 34) experiencing homelessness in Montreal, Canada.

**Methods:**

The qualitative methods we used consisted of semi-structured interviews (*n* = 12) and focus groups (*n* = 2, with a total of 22 participants). The content of the collected data was then analyzed.

**Results:**

Participants identified their various motives for alcohol use (coping with painful memories, dealing with harsh living conditions, socializing/seeking a sense of belonging, enjoying themselves/having fun); their risky drinking patterns (binge drinking, mixing alcohol with drugs, non-beverage alcohol drinking, failing to keep sufficient alcohol on hand to prevent acute withdrawal, drinking in public settings); their harm reduction practices (planning how much to drink, keeping a supply of alcohol to prevent acute withdrawal, hiding to drink, concealing alcohol, drinking alone, drinking/hanging out with others, drinking non-beverage alcohol, and taking benzodiazepines, cocaine or other stimulant drugs); and the rationales underpinning their alcohol use and harm reduction practices.

**Conclusion:**

Associating the drinking motives of a group of study participants with their risky drinking patterns and harm reduction practices shed light on their rationales for alcohol use, yielding insights that could be used to better tailor policies and interventions to their needs.

## Introduction

Broadly available and relatively inexpensive, alcohol is one of the substances most commonly used by people who experience homelessness [29, 62], a population that is also disproportionately affected by the harms associated with alcohol dependence. For example, in a study of women experiencing homelessness across eleven US sites (Health Care for the Homeless clinics), Upshur et al. [72] found rates of alcohol use disorder to be four times higher among the sample than among women who were not experiencing homelessness. Similarly, Fazel et al. [24] put the prevalence of alcohol dependence among people in high-income countries who experience homelessness at roughly 38%, which is about ten times higher than the general population.

Current evidence suggests that alcohol dependence and homelessness are coexisting conditions associated with a wide range of harms. Alcohol dependence among people who experience homelessness has detrimental effects on their physical and mental health (e.g., liver disease, alcohol-related seizures, risk of suicide and depression) [24, 44, 56]. Furthermore, people with alcohol dependence who experience homelessness are likely to use other psychoactive substances, which put them at higher risk of unprotected sex and thus of contracting HIV and/or other sexually transmitted diseases [23, 26, 68]. Psychiatric conditions are also prevalent among this population, the most commonly reported being psychotic and mood disorders [30, 39, 42]. Moreover, alcohol-related mortality among people experiencing homelessness is particularly disproportionate, reaching rates of six to ten times higher than the general population [4]. Lastly, alcohol use can serve as a barrier to health and social services, including primary or specialized health, mental health and housing services [3, 24, 55].

The coexistence of alcohol dependence and homelessness is highly prevalent in Canada. A recent survey suggests that 5% of Canadians have experienced homelessness in their lifetime [9], with addiction or substance use—including alcohol dependence—factoring strongly as a reason for housing loss [20]. Conducted on the night of April 24, 2018, the last count of people experiencing visible homelessness across Quebec identified 5789 such individuals, 54% of whom were in Montreal alone [40]. Yet despite such a high concentration, Montreal lacks emergency housing (e.g., shelters and hostels) allowing alcohol use on-site. Moreover, most shelters refuse to admit people who show signs of alcohol intoxication [49]. The complex interplay between homelessness and alcohol use or dependence thus limits the possibilities of this group in terms of temporary shelter and permanent housing alike.

Both to better understand how people experiencing homelessness engage in risky alcohol use and to develop programs and treatments that target its potential harms, recent research has explored drinking patterns among these populations. Some of these studies use alcohol consumption amounts and/or frequency as their metric for examining the links between drinking patterns and physical health [51], psychiatric disorders and mental health problems [17, 75], housing access and retention [48, 74] and support service engagement [8]. However, few present the perspectives of the drinkers themselves as to what constitutes risky patterns of alcohol use (i.e., drinking patterns that pose a risk to health and well-being) or how they manage the associated risks [14], what Duff [18] terms “informal harm reduction strategies.”

To fill this gap in the literature, our study set out to explore the perspectives of a group of persons experiencing homelessness and alcohol dependence in Montreal, Canada, on what they saw as risky drinking patterns. Participants’ motives for alcohol use as well as the ways in which they prevented or reduced the harms associated with some of these patterns were also examined. In doing so, we highlighted the rationales behind this set of patterns and practices as a means of questioning what at first glance might appear as merely senseless and self-destructive behavior. Our overall aim was to capture participants’ perspectives with a view to better tailoring policies and interventions to their needs and expectations.

## Approach and methods

Our study is a component of a broader feasibility study conducted by an interdisciplinary team at the Institut universitaire sur les dépendances (IUD) and whose aim is to implement services adapted to the needs and expectations of people experiencing alcohol dependence and homelessness in Montreal. The feasibility study’s researchers had decided on the phrase “wet services” as an umbrella term to signify the wide range of arrangements (managed alcohol programs [MAPs], wet shelters, drop-in centers, transitory and permanent housing, etc.) that allow safe indoor alcohol use and, in some cases, offer other services and forms of support [25, 49]. The feasibility study had four objectives: (1) define the wet services offered in Montreal, (2) identify conditions that would favor the implementation of wet services in Montreal; (3) map out the legal and regulatory context specific to Quebec to facilitate the implementation of these services; and (4) develop methods for monitoring and evaluating wet services in Montreal.

The research for this paper, in turn, which was outlined in Objective 2 of the feasibility study, is part of a consultation process conducted with (1) actors outside of Montreal with proven experience implementing wet services; (2) actors in Montreal who work directly with people experiencing homelessness; and (3) people experiencing homelessness and alcohol dependence who could potentially benefit from wet services. Our initial aim had been to gather the perspectives of the latter group about the kinds of wet services that would best address their needs and expectations; however, as our study progressed, we decided to explore their current living conditions, drinking patterns and the associated risks as well.

Participants (*n* = 34) were recruited through purposive sampling. Individuals with profiles corresponding to the potential users of wet services were identified by community organization outreach workers, who then put them in contact with the research team. Those who met the eligibility criteria were subsequently recruited for our study. Candidates referred by word of mouth underwent the same process. The eligibility criteria were: (1) having no fixed address in the last 12 months; (2) self-identifying as being alcohol dependent and engaging in heavy alcohol use [50] and/or binge drinking [71] over the last month, (3) aged 18 or older; (4) able to speak French or English; and (5) able to understand and consent to participate. To ensure sample diversification [60], age, gender and ethnicity were also considered.

The qualitative methods used to capture participants’ perspectives consisted of semi-structured interviews (*n* = 12) and focus groups (*n* = 2, with a total of 22 participants). The interviews thoroughly explored what participants defined as risky patterns of alcohol use and how they managed the associated risks. Certain topics that emerged during the interviews (e.g., motives for drinking and their association to risky drinking patterns) were further explored in two focus groups whose members had characteristics similar to those of the interviewees. By eliciting shared views and disagreements [64], the focus groups shed light on the range of alcohol use experiences within this population. The animated discussions between group participants revealed the contextual dimensions of their drinking practices and broadened the understanding of their motives. Combining semi-structured interviews with focus groups thus helped round out the data by offering insights on a range of individual experiences and contextual circumstances and identifying where the key points of the phenomena under study converged [38].

The interviews and focus groups were led by four professional researchers (three men and one woman) who had masters and/or doctoral degrees in social science and/or public health. All had been professionally trained in the use of qualitative research methods in addition to having extensive and relevant experience conducting this type of research. All had also worked for the IUD and had no pre-existing relationships with the participants. The topics covered in the interview guide included living conditions, drinking patterns and the associated risks, use of services and service needs, as well as how participants might envision future wet services in Montreal. The focus group guide also included such emergent topics as motives for drinking and their association to risky drinking practices. The interviews were conducted in settings familiar to participants (e.g., streets, subway stations, community-based organizations). Though some of these settings were public, only the participants and researchers were present during the interviews. The focus groups were held in private rooms at the community-based organizations that had supported the recruitment process. All of the interviews (20 to 80 min long) and focus groups (75 to 100 min long) were recorded. Participants were offered monetary compensation of $40 CAD for their time and input.

The audio recordings of the interviews and focus groups were digitally transcribed. For privacy reasons, names and all additional identifying information were anonymized. Data from the interviews and focus groups were integrated in a single data set. Content analysis [13] was performed by three researchers (RMO, NIS, JFA), who read and codified the data using NVivo 12 software. The researchers did not pre-define any coding categories, but rather let the codes emerge from the data [33]. To resolve disagreements about coding and reach consensus, researchers met in person during the analysis (for at least seven two-hour meetings). Data integration yielded overlapping and richly complementary findings that afforded a more nuanced understanding of drinking motives, risky drinking patterns and harm reduction practices [38]. Particular attention was paid to the connections established by participants between these three parameters, which often emerged as they described their difficult living conditions and how they coped with environmental risks. Lastly, to ensure validity, the researchers met to analyze over 10% of the gathered data, reaching 95% agreement [45].

## Results

This section presents information on participants’ sociodemographic characteristics. Their motives for alcohol use, risky drinking patterns, harm reduction practices and drinking/harm reduction rationales will also be examined.

### Sociodemographic information

Over half of the participants were men, two-fifths were women and one-tenth identified as outside the gender binary. They were aged between 24 and 71, with a median age of 49.4 years. Almost all were born in Canada (half of these, in the province of Quebec); and over half of the group spoke French as a first language. Four-fifths identified as Caucasian and one-fifth, as Indigenous (First Nations, Inuit or Métis). All identified as heavy alcohol users and being alcohol dependent. All drank alcoholic beverages; one-fifth also consumed non-beverage alcohol. “Non-beverage alcohol” refers to liquids with extremely high alcohol concentrations—mouthwash, hand sanitizer, etc.—that are not intended for human consumption but are used instead of beverage alcohol for reasons of affordability and accessibly [14, 54]. Most participants used substances other than alcohol as well, the most common being cannabis, cocaine (powder and crack cocaine), amphetamines, prescription opioids (hydromorphone being the most commonly used, known in Canada by the commercial name of Dilaudid) and benzodiazepines (clonazepam and lorazepam being the most commonly used, known in Canada by the commercial names of Rivotril and Ativan). All participants identified as either currently experiencing homelessness or as lacking stable, safe and adequate housing. They accommodations alternated between their own apartments or rooms and transitional housing, friends’ apartments, hotel rooms and the street. All had lived on the street for periods ranging from one month to 15 years.

### Motives for alcohol use

To gain a deeper understanding of participants’ risky patterns of alcohol use, we explored how these might intersect with their motives for drinking. A wide range of motives emerged, the most common being coping with the harsh conditions of life on the streets, dealing with painful memories, wanting to socialize and be part of a group, and looking for fun and good times.

The first of these—coping with the harsh conditions of the streets—was the most commonly raised. A number of participants said they drank to “warm up,” i.e., to better endure Montreal’s cold climate. Some also mentioned street noise, saying the “only way to sleep” was by “drinking a few beers.” Cocaine and other stimulant drugs (e.g., speed, methamphetamine), in turn, prevented them from getting “too drunk” and being exposed to the dangers of the streets (e.g., robbery, aggression, police harassment). Others said they combined alcohol with drugs to “escape reality” and forget that they did not know “where to live,” were “poor” and were “seen as trash” by most people. As Mario reported:

When you are homeless, you always want to run away from who you are, and the beer is always there to help you to run away from you. Beer helps you to forget the life you have, your problems, your sh*tty life! Beer puts you in trouble too; it does not fix anything at the end [laughs]. (Focus group)

Most participants also said they used alcohol to cope with painful memories. Some described drinking as a strategy to “hide what had happened in the past” or “freeze emotions,” linking alcohol use to traumatic events. Childhood memories of abuse, domestic violence and/or loss of loved ones remained vivid for many, as did the memories of being victimized while living on the street. Alcohol and, in some cases, drugs helped them “move away” from such memories and function in the day-to-day. Cedric, for example, described alcohol as his “medicine to forget the past.” (Focus group).

Socializing with others who lived on the street was a further motive. Alcohol in this sense was integral to the “homeless culture”; participants drank to make friends, be part of the group and feel a sense of community. Regularly drinking with friends served to strengthen relationships of mutual support. As noted by Fatima, “having friends to protect your back” was strategic “to surviv[ing] on the streets.” (Focus group).

For some, a significant motive for drinking was the quest for fun and “good times.” Arthur said that, “like everyone else,” he liked to “share and party” with friends, particularly to mark moments like anniversaries, holidays or being paid. On these occasions, participants underscored that they were in a “celebratory mood” and would drink “a lot” or binge drink. Ariel described what she and her friends would do on payday:

We often do a big party with friends. Of course, it’s fun; but you know sometimes we drink too much and we spend all our money. Anyway, at least we celebrate that we are alive. (Semi-structured interview)

### Risky patterns of alcohol use

Participants described five drinking patterns they saw as potentially dangerous or posing a risk to their health and well-being. These were: (1) binge drinking; (2) mixing alcohol with other drugs; (3) drinking non-beverage alcohol; (4) not keeping a sufficient alcohol supply to prevent withdrawal symptoms; and (5) drinking in public. Participants also described how they reduced the harms associated with some of these patterns.

#### Binge drinking

Most participants engaged in binge drinking, ingesting large quantities of alcohol over a short period of time. As mentioned above, heavy drinking would often occur on special occasions that revolved around having a good time with friends. However, some remarked that they often couldn’t stop, once they’d started. Martha defined her alcohol use as follows:

I’m a very alcoholic person. I spend all my money on beer at the five-dollar pocket dépanneurs [convenience stores]. When I start drinking, I can’t stop. I go to the dépanneur and buy cheap beer. I don’t stop until blackout. A real alcoholic, f*ck! (Focus group)

Some participants said that, to avoid binge drinking, they planned how much they were going to drink on a given day, then tried to buy only the intended amount of alcohol. Others preferred to “drink alone,” thus avoiding peer pressure to drink copiously and remain “in control” of how much they drank (semi-structured interview). When they drank “too much” and wanted to sober up, they smoked crack, injected cocaine or took other stimulant drugs.

#### Mixing alcohol with drugs

Most participants consumed alcohol with other drugs, cannabis being the most frequent, followed by cocaine (powder and crack) and opiates (heroin and hydromorphone). Several identified as “poly-consumers” or “poly-addicted” people, i.e., who were dependent on more than one substance and had to simultaneously manage multiple addictions. For example, Armand stated that he was addicted to alcohol, heroin and cocaine and that his daily challenge was to come up with the cash to buy all three. (Semi-structured interview).

The majority reported purposely mixing alcohol with other substances to either reduce the undesirable effects of the former or potentiate its desirable aspects. Marie, for example, felt that alcohol rendered users more hostile.

Alcohol makes me aggressive. Alcohol is the substance that makes people more aggressive. When I feel like everyone’s starting to get on my nerves, I grab a few puffs of pot, a joint, and it calms me down. I feel more at peace, relaxed when I smoke pot. It let me relax, enjoy the moment. (Focus group)

To enhance alcohol’s desirable aspects (its relaxing and soothing effects), some participants took opioid-based drugs, either street drugs such as heroin or prescription opioids such as hydromorphone. They would often do this when, as Antoine said, they wanted to “erase [their] past” temporally and “forget” about their current “homeless life.” (Semi-structured interview).

#### Non-beverage alcohol drinking

The frequent use of non-beverage alcohol—a substance both affordable and accessible—by people who experience homelessness has been extensively reported in the literature (e.g., [19, 21, 76]). In Canada, its consumption is widespread among these populations and its harmful effects, a matter of concern [7, 27]. However, most participants reported that they did not drink non-beverage alcohol. Indeed, participants in one focus group said that nobody in Montreal drank non-beverage alcohol because beer was extremely cheap:

Lucy: Here, we can get a beer for a dime and a half. Easily, we can find a dollar and a half, and we get a beer . . . It’s so cheap! There is even a place that sells the beer at one and thirty-five cents.

Marie: We’re not in Russia here.

Sandra: [People] here don’t drink [non-beverage alcohol] because Montreal has the cheapest booze in all of Canada! It’s infamous for being cheap with the booze . . . In other cities, people drink it all the time.

However, various interviewees reported having seen “a friend” or “someone on the streets” drinking mouthwash, hand sanitizer or rubbing alcohol. Only a small minority admitted to drinking non-beverage alcohol in extreme situations when they were unable to buy or access alcohol and wanted to prevent withdrawal symptoms. Some participants reported drinking home-distilled alcohol (e.g., moonshine and *baboche* [artisanal alcohol made from organic materials]) while they were incarcerated. It is possible that the strong stigma associated with drinking non-beverage alcohol may have hindered a more open discussion as to its use; the practice may therefore be more prevalent than what was reported.

#### Not keeping alcohol on hand to prevent withdrawal symptoms

Most participants said that, due to their heavy drinking habits and alcohol dependence, they usually awoke with withdrawal symptoms. These included shaky hands (“the shakes”), headache, vomiting, rising heart rate, sweating, insomnia and seizures (“the bacon”). Frédéric commented:

You know, there are people who need a coffee to start the day, but for me it’s alcohol. If I don’t have my alcohol dose, two beers, very fast, when I wake up in the morning, I become sick, I start shaking like a leaf, then it’s not pretty, it’s not funny at all. (Semi-structured interview)

Other participants also reported being hospitalized due to seizures and heart complications. To prevent alcohol withdrawal, they usually kept a backup supply on hand. For example, Arleen said that, every night before going to sleep, she stopped by a *dépanneur* (convenience store) to buy “a big beer can, 10% alcohol” that she didn’t “touch over night” and drank only “first thing in the morning.” (Semi-structured interview) While Montreal convenience stores generally open at 9 a.m., participants often awaken with withdrawal symptoms earlier.

However, several participants said that, due to their precarious and unstable living conditions as well as their binge-drinking practices, they often lacked the resources to obtain alcohol to prevent withdrawal. When this occurred, some said they resorted to benzodiazepines (e.g., clonazepam or lorazepam) to “cut off the shakes” and other symptoms. Interestingly, they also took benzodiazepines when they wanted to “get rid” of withdrawal symptoms and “be able to function” without alcohol. For example, Antione stopped drinking and “did three days of Rivotril [clonazepam]” before visiting his son, who was at the Direction of Youth Protection (DYP). Antoine, who had feared the DYP social worker would forbid him from seeing his son if he showed signs of alcohol intoxication, said: “The Rivotril did the job. I didn’t shake, I didn’t feel sick at all.” (Semi-structured interview).

#### Drinking in public settings

All participants reported regularly drinking in public places (e.g., parks, street corners, parking lots, subway stations); some felt that, as street people, they had no choice. Participants reported experiencing stigmatization and discrimination at such times. Passers-by sneered with “judgemental eyes,” “moved away” from them and made insulting comments about their alcohol use. Furthermore, the accusations often leveled against the Montreal police—i.e., that people experiencing homelessness were subject to social profiling—were confirmed by participants, who reported having their alcohol confiscated by officers or being forced to “pour it out,” as well as having their sobriety “tested” in questionable ways (e.g., being ordered to walk a straight line). Furthermore, the police also constantly handed out “tickets” for drinking or being inebriated in public, as described by Cédric:

I have accumulated so much debt in unpaid tickets that it’s almost the price of a car f*ck! If you [the researcher] and your friends drink beer in a park, the cops won’t tell you anything, but we, they piss us off with tickets. I have an astronomical debt; I would never be able to pay it [off]. (Focus group)

To avoid stigmatization, discrimination and harassment, most did their drinking in more secluded areas, i.e., alleyways, parking lots or the less-frequented parts of subway stations. They also concealed their alcohol in non-alcoholic beverage bottles and disposable coffee cups. Some participants like Armand said that “drinking alone” was a “form of protection,” since it attracted less attention from law enforcement, thus reducing the “chances” of being “caught drinking.” (Semi-structured interview) However, others said the opposite, i.e., that to protect themselves from aggression and “feel secure,” they needed to “hang out” with friends. Some also reported having dogs, not just for company, but for protection as well. Sandra, for example, said:

On the streets, you get drunk to stay warm and then you get vulnerable because you are drunk. I had my dog by my side, and he became aggressive because [of protecting me from] people who are f*cking sickening. I’ve almost gotten stabbed in my sleep for no reason. (Focus group)

### Rationale of alcohol use and harm reduction

**Fig. 1:**
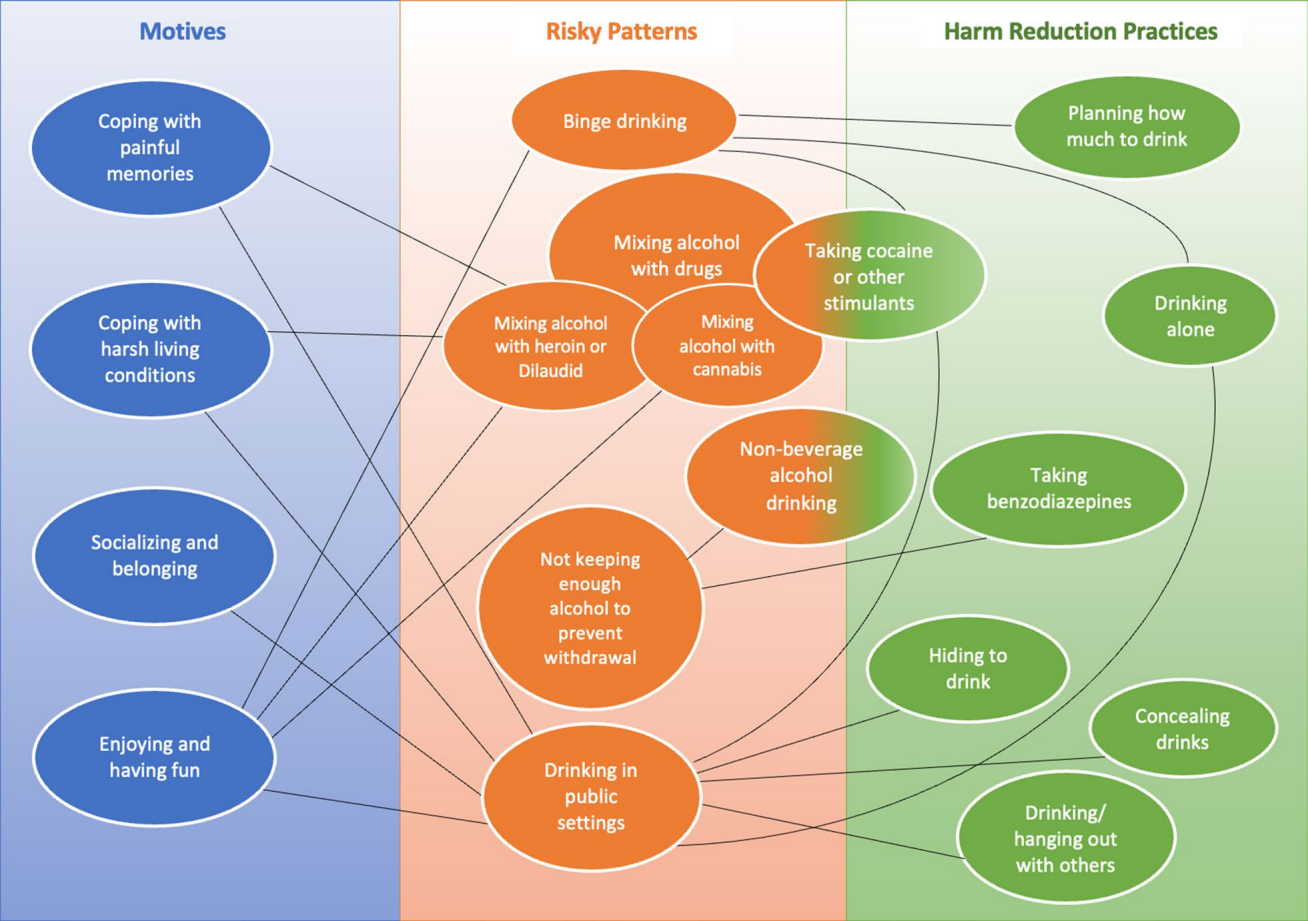
Motives for alcohol use, risky drinking patterns and harm reduction practices

To this point, we have presented the drinking motives, risky drinking patterns and harm reduction practices reported by participants. Below, in Fig. 1, we summarize these and highlight their associations to reveal the rationales behind participants’ use of alcohol and other substances as well as their management of the perceived harms. We are not claiming that these are the only possible associations—there are plenty of others—but merely presenting them as the ones raised by our sample.

Participants’ motives for alcohol use are associated with three of their five risky drinking patterns. Coping with painful memories and with harsh living conditions correlate to mixing alcohol with heroin or hydromorphone; enjoying and having fun with others, to binge drinking and to mixing alcohol with drugs (opiates and/or cannabis); and all four motives, to drinking in public settings.

Participants also enacted eight harm reduction practices to lessen the risks associated with three of the five risky patterns. To prevent binge drinking, participants planned how much they intended to drink or drank alone; to sober up after engaging in binge drinking, they took cocaine or other stimulants. To reduce the risks associated with drinking in public settings, they concealed their drinks, hid to drink, drank alone, hung out with others while drinking or took cocaine or other stimulants to get sober. To ward off withdrawal symptoms when they had no backup alcohol supply, they drink non-beverage alcohol or took benzodiazepines. It should be noted that, while that keeping sufficient alcohol on hand to prevent withdrawal is itself a harm reduction practice, it has not been included in Fig. 1, since participants had defined being unable to prevent withdrawal in this way as a risky drinking pattern.

Several of our participants’ harm reduction practices—e.g., planning how much to drink, keeping enough alcohol on hand to prevent withdrawal symptoms, hiding to drink and concealing their drinks—effectively minimize the negative health, social and legal impacts associated with alcohol use. Nonetheless, from our standpoint, some of their harm reduction practices may entail risks to health and well-being. Taking cocaine after drinking, for example, increases the toxic effects of both substances, which can produce severe side effects such as overdose, stroke, heart attack or liver damage [58]. Drinking non-beverage alcohol, in turn, poses serious risks of injury, disease and even death due to the high alcohol concentrations in these liquids [76]. While taking benzodiazepines to avoid or reduce alcohol withdrawal symptoms is considered a safe evidence-based detoxification strategy [65], abrupt cessation of benzodiazepines can have side effects like sleep disturbance, increased anxiety, panic attacks, headache, muscular pain and stiffness, or seizures [59]. Finally, drinking alone to avoid being targeted by police officers turns participants into easy targets for aggression and robbery from others, while hanging out with other drinkers for protection increases their visibility and, consequently, their vulnerability to police harassment.

## Discussion

Our study aimed to explore risky patterns of alcohol use among a group of persons experiencing alcohol dependence and homelessness in Montreal. To further understand these patterns, we have also examined participants’ motives for alcohol use as well as the ways in which they manage the associated harms. Our goal in this was to contribute to the growing literature on patterns of alcohol and/or substance use among persons who experience homelessness. Various studies have documented alcohol use patterns in combination with drugs (cocaine, cannabis, and/or heroin) among people experiencing homelessness as risk factors for unprotected sex [23, 26] and HIV transmission [37, 68]. Incidences of heavy alcohol use and binge-drinking patterns, particularly among runaway and street youth [43, 52, 67], have also been well documented, along with the prevalence of non-beverage alcohol drinking among people experiencing homelessness when they cannot afford regular alcohol [21, 22, 61, 69, 76]. However, to our knowledge, few studies [70] have examined how the particular contexts in which these persons drink shape their drinking patterns and constitute a significant source of harm. To our thinking, the focus for harm reduction interventions aimed at people experiencing alcohol dependence and homelessness should not be the individual, but rather the risks associated with their environment and how these might be mitigated. As previously pointed out, in Montreal there is a concerning absence of emergency housing allowing alcohol use on-site as well as a limited number of wet services [25]. The implementation of wet services (MAPs, wet shelters, drop-in centers, transitory and permanent housing, etc.) that provide opportunities to drink safely should therefore be considered a priority in terms of harm reduction.

Our study has set out to inform the policies and interventions oriented toward people experiencing alcohol dependence and homelessness by examining the overlap between drinking motives, risky drinking patterns and harm reduction practices. Crucially, we have also explored the rationales underpinning these behaviors, which might otherwise be misconstrued as merely senseless and self-destructive. We propose that identifying the motives for alcohol use and their associations with risky drinking patterns can lead to interventions that address the root causes of such patterns. For example, if people who experience homelessness mix alcohol with heroin or hydromorphone and drink in public settings to cope with painful memories and harsh living conditions, then interventions that provide housing and psychological/psychiatric services along with alcohol would be more apt to positively impact the health and well-being of these persons, as the literature on MAPs has shown (e.g., [56, 61, 69, 73]). Similarly, identifying the relationships between risky drinking patterns and harm reduction practices has brought to light the additional risks posed by such practices—for example, failing to make provisions for alcohol withdrawal or attempting to prevent it by drinking non-beverage alcohol and taking benzodiazepines, all of which could seriously compromise health. The COVID-19 pandemic and its repercussions, including the associated public health response of social distancing and physical isolation and the accompanying cuts to essential services, also served to complicate participants’ access to legally regulated alcohol. In this scenario and in line with the safe supply programs developed for opioid-dependent persons (e.g., [6, 15, 34]), we propose harm reduction initiatives that see alcohol provision as necessary to protecting the health of people experiencing alcohol dependence and homelessness. In addition to encouraging the replicability of MAPs, another move in this direction would be to allow services for people experiencing homelessness to offer their users regulated alcohol doses when they are at risk of alcohol withdrawal.

We also attempted to shed light on the complex motives underpinning alcohol use and risky drinking practices among persons who experience homelessness. The reasons discussed in the extensive literature on the motives for drinking in various populations—college students, teens, veterans, people experiencing homelessness, runaway youth, people with adverse childhood experiences and/or who experienced trauma, women in vulnerable situations, etc.—include the desire to cope with trauma, escape problems, self-medicate for mental health conditions, get drunk, conform, socialize and celebrate (e.g., [12, 16, 31, 37, 53, 63, 66]). Despite this diversity, some researchers [1, 13] broadly divide motives for alcohol use into two categories: coping with negative emotions (e.g., escaping trauma/problems) and enhancing positive emotions (e.g., socializing, celebrating). Most research on drinking motives among homeless adults has focused on the first category, offering valuable insights into how drinking to cope mediates the relationship between psychiatric symptoms and consumption [77] and/or trauma and victimization [5, 35, 41]. Our own findings on drinking to cope with harsh living conditions and painful memories are consistent with this. However, participants in our study also reported drinking for reasons that correspond to the second category of motives, including socializing, feeling a sense of belonging and having fun. Moreover, they linked these motives with risky drinking patterns such as binge drinking and drinking in public settings. Drinking to socialize, be “part of the gang” and have fun highlights the importance of alcohol in the social fabric of these individuals—not a surprise, given that most of them are alienated from their families and communities. Our research therefore points up the need for interventions focused on creating and maintaining strong, healthy social relationships that provide a sense of belonging, pleasure and enjoyment. Although further work in this area is needed, research on programs like Housing First has shown that strong working relations between staff and tenants are crucial for the harm reduction to be effective [36], and that strengthening positive relationships in tenants’ social networks (mostly with staff and family members) is beneficial to recovery [28] and subjective well-being [2]. It has also been reported that when MAP users stabilized their alcohol intake, they reconnected with family members and friends, with positive impacts on their well-being [56, 57].

Part of our findings on participants’ practices to reduce the risks associated with their alcohol use align with the harm reduction strategies advanced by government and community-based organizations, such as planning how much to drink and drinking with trusted others (e.g., [10, 11, 32, 46, 47]). These recommendations are mostly preventive and focused on groups (adolescents, youth, seniors) at risk of developing alcohol dependence, excluding those who already experience alcohol dependence and vulnerable living conditions. However, our bottom-up approach highlights harm reduction practices that echo the Safer Drinking Tips infographics created by people with lived experienced (Eastside Illicit Drinkers Group for Education) and published by the Canadian Institute for Substance Use Research (https://www.uvic.ca/research/centres/cisur/projects/map/index.php). These infographics provide accessible information tailored to the risk management needs of people experiencing alcohol dependence and homelessness in the Vancouver area. We recommend implementing a similar approach in Montreal, but adapted to the particularities of the local context. As our research shows, some of the harm reduction practices used by our participants (e.g., hiding to drink, concealing their alcohol) are shaped by the lack in Montreal of safer places to drink. Such an approach should also raise awareness about the unintended risks posed by a number of these harm reduction practices.

Our study’s findings must be interpreted within the context of various limitations. Firstly, our research was based on qualitative methods that could lead to a social desirability bias, causing participants to provide answers they felt would be favorably viewed by the researchers. However, we believe this limitation is outweighed by the advantages of these methods (semi-structured interviews/focus groups), inasmuch as they allow us to grasp the perspectives of persons experiencing alcohol dependence and homelessness. Secondly, we could have used other qualitative methods such as participant observation to triangulate what our participants reported with what they did, which would have been useful for further exploring topics such as their use of non-beverage alcohol. But since our goal was to examine their experiences of alcohol use, we considered semi-structured interviews and focus groups as the most appropriate methodological choice. Thirdly, the recommendations that emerged from our findings may have limited scope, given that the coexisting issues of alcohol dependence and homelessness have a particular configuration in Montreal. For example, the non-beverage alcohol use that has been identified as an issue of concern elsewhere in Canada would seem to have minor implications for most of our participants, who said they didn’t drink it due to the low cost of beer in Montreal. Nonetheless, our description of the research context may allow the reader to identify recommendations that may work under similar conditions. Lastly, no diagnostic criteria were used to determine whether the participants had psychiatric conditions or further explore the associations between their motives for drinking and their risky drinking patterns. Rather, participants had been asked to self-report on their mental health and alcohol dependence.

### Recommendations for practice and policy

Our findings show how diverse elements from a specific context influence the drinking patterns of a group of people experiencing homelessness and alcohol dependence and increase the risks associated with such patterns. In general, our study highlights the urgent need to increase the availability of wet services—where people experiencing homelessness and alcohol dependence can safely drink as well as receive shelter and support—in Montreal. Furthermore, our findings on certain alcohol use motives (e.g., coping with painful memories and with the harsh conditions of life on the streets) point to the need for interventions that address the psychological and social problems at the root of risky drinking patterns. Findings on other motives (e.g., socializing, belonging to a group, enjoying good times and having fun with others), in turn, illustrate alcohol’s relevance to social relations among people experiencing homelessness and alcohol dependence. Interventions aimed at creating and maintaining strong and healthy social relations may thus reduce the role of alcohol in forging social bonds. Moreover, the recurring descriptions of the participants of the injurious effects of alcohol withdrawal highlight the need for policies and regulations that would enable services to offer regulated alcohol doses to users who are at risk of withdrawal. Finally, given the additional health risks posed by some of the harm reduction practices reported (e.g., taking cocaine or other stimulants, drinking non-beverage alcohol, taking benzodiazepines, drinking alone, and drinking/hanging out with others), we suggest that accessible documents—pamphlets, infographics, posters—on safe drinking strategies, informed by the experiential knowledge of persons experiencing homelessness and alcohol dependence and adapted to their particular contexts, be created and disseminated.

## Conclusion

In this paper, we have explored the motives for alcohol use, the risky drinking patterns and the harm reduction practices of a group of individuals experiencing alcohol dependence and homelessness in Montreal, Canada. The main drinking motives reported by study participants were coping with painful memories, coping with harsh living conditions, socializing and getting a sense of belonging, and enjoying good times and having fun. Their reported risky drinking patterns were binge drinking, mixing alcohol with drugs, non-beverage alcohol drinking, not keeping enough alcohol on hand to prevent withdrawals and drinking in public settings. They also engaged in practices aimed at reducing the harms associated with their alcohol use, such as planning how much to drink, keeping a supply of alcohol to prevent withdrawal symptoms, hiding to drink, concealing their alcohol, drinking alone, drinking/hanging out with others, drinking non-beverage alcohol, taking benzodiazepines and taking cocaine or other stimulant drugs. The associations between motives for alcohol use, risky drinking patterns and harm reduction practices show the rationales underpinning participants’ alcohol use and risk management. In using qualitative methods to capture the perspectives of people experiencing alcohol dependence and homelessness, our overall aim was to better inform policies and interventions and improve how these are tailored to the participants’ specific needs and expectations.

## Data Availability

The database created and analyzed for this study is not publicly available. It is based on a small sample of research participants, which threatens their confidentiality. It could be available from the corresponding author subject to reasonable request.
